# Magnetic Nanofiber Mats for Data Storage and Transfer

**DOI:** 10.3390/nano9010092

**Published:** 2019-01-12

**Authors:** Christoph Döpke, Timo Grothe, Pawel Steblinski, Michaela Klöcker, Lilia Sabantina, Dorota Kosmalska, Tomasz Blachowicz, Andrea Ehrmann

**Affiliations:** 1 Faculty of Engineering and Mathematics, ITES, Bielefeld University of Applied Sciences, 33619 Bielefeld, Germany; christoph.doepke@fh-bielefeld.de (C.D.); timo.grothe@fh-bielefeld.de (T.G.); michaela.kloecker@fh-bielefeld.de (M.K.); lilia.sabantina@fh-bielefeld.de (L.S.); 2 Institute of Physics—Center for Science and Education, Silesian University of Technology, 44-100 Gliwice, Poland; psteb@bobolin.com.pl (P.S.); dorotakosmalska95@gmail.com (D.K.); tomasz.blachowicz@polsl.pl (T.B.); 3 Faculty of Electronics and Informatics, Koszalin University of Technology, 75-453 Koszalin, Poland

**Keywords:** electrospinning, magnetic nanoparticles, nanofiber mat, neuromorphic computing, micromagnetic simulation, magnetic signal transfer, magnetic switch, Magpar

## Abstract

Electrospun nanofiber mats may serve as new hardware for neuromorphic computing. To enable data storage and transfer in them, they should be magnetic, possibly electrically conductive and able to respond to further external impulses. Here we report on creating magnetic nanofiber mats, consisting of magnetically doped polymer nanofibers for data transfer and polymer beads containing larger amounts of magnetic nanoparticles for storage purposes. Using magnetite and iron nickel oxide nanoparticles, a broad range of doping ratios could be electrospun with a needleless technique, resulting in magnetic nanofiber mats with varying morphologies and different amounts of magnetically doped beads.

## 1. Introduction

Magnetic nanofibers are of large interest for basic research of their magnetic properties as well as for possible applications in spintronics or neuromorphic computing. Several groups have investigated possibilities to prepare them. Typical methods to create magnetic nanofibers include vapor growth [1], anodizing processes [2], e-beam lithography [3], focused-ion-beam milling [4] or template-based methods [5].

Another possibility to unambiguously create nanofibers is electrospinning. This technology can be used to create nanofiber mats from different man-made polymers [6,7,8], biopolymers [9,10,11] or polymers blended with nonsolvable materials [12,13,14].

Most recently, Liu et al. showed that magnetic iron acetylacetonate could be needle-electrospun with polyacrylonitrile (PAN) dissolved in dimethyl formamide (DMF), and afterwards stabilized and carbonized, resulting in Fe_3_C/N-C nanofibers [15]. Na et al. created magnetic ferrite nanofibers in a polyvinyl alcohol fiber matrix and calcinated the polymer afterwards to obtain pure magnetic nanofibers [16]. Lin et al. reported about carbonized PAN/cobalt ferrite nanofibers as a catalyst [17].

Other approaches concentrate on electrospinning pure polymer nanofibers and afterwards adsorbing magnetic nanoparticles on the fiber surfaces, e.g., for magnetic hyperthermia applications [18] or electromagnetic shielding [19]. However, in this way, the magnetic nanoparticles cannot be embedded inside the nanofibers which will lead to other magnetic properties.

The magnetization dynamics and quasistatic magnetic properties of such nanofibers were investigated theoretically and experimentally by different groups [20,21,22], indicating the large influence of the material, the fiber diameter and especially the orientation with respect to the external magnetic field on their magnetic properties [23,24]. It should be mentioned that opposite to common integrated circuits, hardware for neuromorphic computing needs a certain statistic approach, suggesting that randomly oriented nanofiber mats will show more interesting properties than perfectly aligned fibers with identical diameters and without bending radii. This is why no setup was chosen in which the fibers were aligned, as discussed in diverse papers [25,26].

For their use in possible future applications in neuromorphic computing, magnetic nanofibers—which could be used to transport data in the form of domain walls moving along them—should be combined with objects that could be used to store data. Typically, memristors or other relatively complicated elements are included in such networks that are planned to be used for neuromorphic computing [27,28,29]. However, this cannot be realized in a one-step electrospinning process. The easiest approaches to realize a combination of nanofibers with a data storage opportunity by electrospinning are preparing either combinations of nanofiber mats with embedded nanomembranes, which can simply be realized by decreasing the distance between the high-voltage electrode of the electrospinning apparatus and the substrate on which the fibers are placed [30], or creating combinations of nanofiber mats with embedded beads, which happens automatically when the polymer solid content in the spinning solution is reduced, as compared to ideal values [31]. Here we report on the second method.

From simulations and experiments it is well-known that nanocylinders or beads with diameters of several hundred nm are in a so-called vortex state for small or vanishing external magnetic fields [32]. This means that the magnetization rotates in a closed loop, while it points in one of the directions perpendicular to this loop in the middle, the so-called vortex-core. This vortex core has two possible orientations and is thus often suggested as a possibility to store data. The vortex core can be switched between both orientations by short magnetic field pulses perpendicular [33,34,35] or parallel to the plane of the vortex [36]. Investigations of the static [24] and dynamic magnetization behavior of the nanofibers between the beads [37] were performed to evaluate the possibilities to switch the beads in nanofiber mats by magnetic field pulses as a prerequisite of the experimental investigations depicted here. In the results, we will show a few simulation results underlining that the beads can even be used as switches which are basic elements of logic circuits.

Magnetic nanofiber mats with magnetic beads were prepared using PAN blended with two different magnetic nanoparticles in diverse concentrations. Our results show that not only the nanoparticle concentration, but also the magnetic material influences the nanofiber mat morphology and distribution of magnetic particles.

## 2. Materials and Methods

The polymer part of the spinning solution consisted of 14% polyacrylonitrile (X-PAN, Dralon, Dormagen, Germany) dissolved in DMSO (min 99.9%, purchased from S3 chemicals, Bad Oeynhausen, Germany). This material was chosen because it can be stabilized and carbonized in future experiments to make it also conductive [38]. The following magnetic nanoparticles were added: Fe_3_O_4_ (magnetite) with particle size 50–100 nm, and Fe_2_O_3_/NiO (diiron nickel tetraoxide) with particle size < 50 nm (both purchased from Merck KGaA, Darmstadt, Germany). Both materials are known to be ferrimagnetic as bulk materials and may become superparamagnetic in small enough nanoparticles [39,40]. The polymer:nanoparticle weight ratios under examination in this study were 1:1.8, 1:1.25; 1:0.79; and 1:0.38, corresponding to the range of spinnable solutions resulting in nanofiber mats which are attracted by weak magnets. All solutions were prepared by stirring the polymer solution for 2 h on a magnetic stirrer, adding the magnetic nanoparticles and stirring manually for 10 min before the nanoparticles were dispersed in an ultrasonic bath for 40 min at 35 °C with a frequency of 37 kHz.

**Figure 1:**
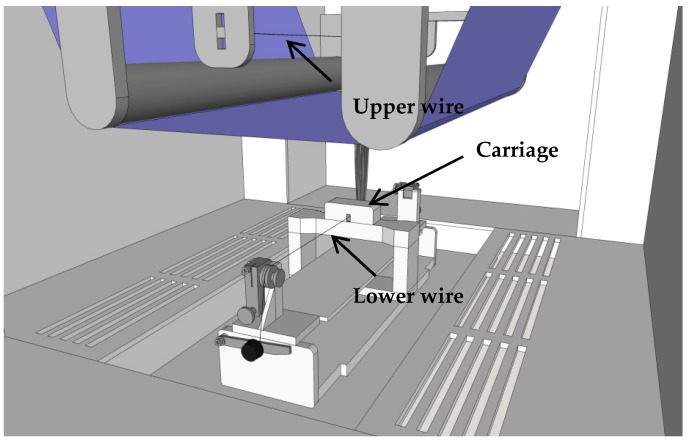
Sketch of the Nanospider. The carriage containing the spinning solution coats the lower electrode wire through a spinning nozzle. A high electric field drags the polymer coating to the upper electrode, in this way elongating and thinning the polymer droplets until they become nanofibers and are finally stopped on the substrate (blue).

Nanofiber mats were created with the needleless electrospinning machine “Nanospider Lab” (Elmarco, Liberec, Czech Republic) on a polypropylene nonwoven as substrate. The function of the Nanospider is depicted in Figure 1. The following spinning parameters were used: high voltage 80 kV, nozzle diameter 0.9 mm, carriage speed 150 mm/s, ground-substrate distance 240 mm, electrode-substrate distance 50 mm, temperature in the chamber 22 °C, relative humidity in the chamber 32%. In all tests, a spinning solution of 5 mL was used which was electrospun in ~13 min.

For the optical examination of the nanofiber mats, a confocal laser scanning microscope (CLSM) VK-9000 (Keyence, Neu-Isenburg, Germany) with a nominal magnification of 2000× was used. An atomic force microscope (AFM) FlexAFM Axiom (Nanosurf, Liestal, Switzerland) as well as a scanning electron microscopy (SEM) Zeiss 1450VPSE were applied for more detailed examinations of the fiber surfaces and diameters, the latter being calculated using ImageJ 1.51j8 (from National Institutes of Health, Bethesda, MD, USA).

**Figure 2:**
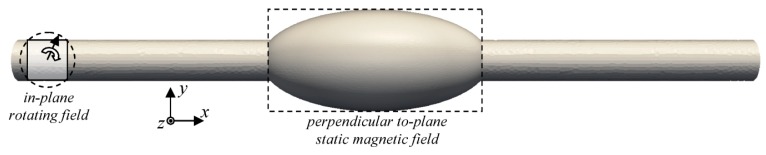
Magnetic nanofiber with bead, simulated using a static and a rotating magnetic field. The fiber diameter is 200 nm, the length 3600 nm, the cross-section diameter of the bead is 560 nm and the bead length 1120 nm.

Micromagnetic simulations were performed using the micromagnetic solver Magpar [41], dynamically integrating over the Landau-Lifshitz-Gilbert equation of motion. The simulation parameters are: magnetic field rotation frequency = 0.5 GHz, rotating field amplitude *B* = 1 T; static field amplitude 0 T or 0.1 T; anisotropy constants K1 = K2 = 0, saturation polarization *J_S_* = 1.0053 T, effective exchange constant *A* = 1.3·10^−11^ J/m, Gilbert phenomenological damping constant α = 0.02 (all values similar to permalloy material parameters). The simulated geometry is depicted in Figure 2.

## 3. Results and Discussion

**Figure 3:**
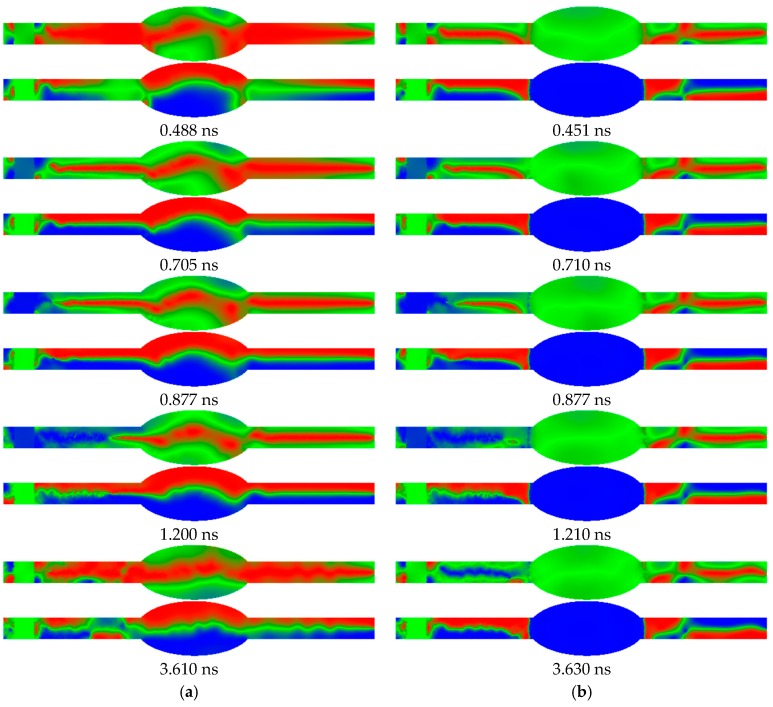
Time-resolved micromagnetic simulation of a bead on a nanofiber: (**a**) Without static magnetic field; (**b**) with static magnetic field of B = 0.1 T along the -z direction applied at the bead. In each pair of images, the upper one represents the x-magnetization component, the lower one the magnetization in z-direction (cf. Figure 2). Color code: red = parallel to the respective axis; blue = antiparallel; green = perpendicular.

Firstly, in order to show why electrospinning nanofiber mats with beads on the nanofibers, Figure 3 depicts two exemplary micromagnetic simulations of such a system.

In both cases, a signal moves from the left end of the system to the right side. While Figure 3a shows the situation without a static magnetic field applied at the bead, a relatively small field of *B* = 0.1 T is applied in the bead region. In case (a), the z-components of the beads are split in red and blue halves, showing that the beads are in a vortex state with the magnetization rotating around the *x*-axis (i.e., the fiber axis). In case (b), however, the magnetization of the bead is fully oriented along the z-orientation.

These different magnetic states of the beads result in different signal transfer behavior. While the bead in the vortex state transfers the signal in the shape of a snake-like gyrotropic precession of the vortex core in the nanofiber on the right side of the bead after 3.6 ns (Figure 3a), the right side stays unchanged if the bead is in the state with aligned magnetization. In this way, signal transfer through a bead can be switched on and off. As this example shows, depending on the choice of materials and bead dimensions, not only storing data in beads is possible, as already described in the literature, but also using them as switches and other logic elements like AND or NAND [37].

**Figure 4:**
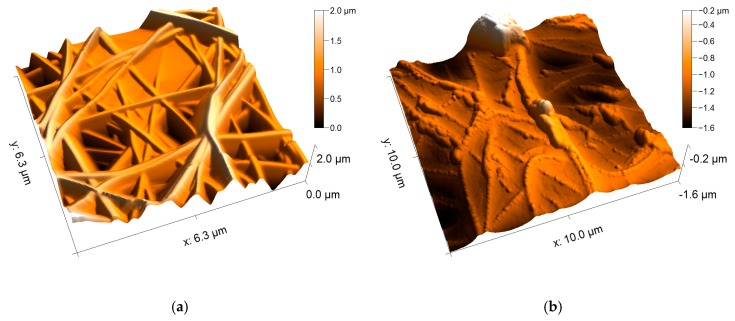
AFM (atomic force microscopy) images of PAN (polyacrylonitrile) nanofiber mats: (**a**) pure PAN; (**b**) pure PAN dip-coated in magnetite nanoparticle dispersion and dried at air.

Experimentally it was tested whether AFM images would reveal nanoparticles outside fibers, or whether a dense layer of magnetic nanoparticles surrounding a nanofiber could be misinterpreted as a pure nanofiber with increased surface roughness. Figure 4 shows a comparison between a typical pure PAN nanofiber mat and the same mat after dip-coating in magnetite nanoparticle dispersion and drying at air. Apparently, adsorbed nanoparticles are clearly visible on the nanofiber surface (Figure 4b), thus AFM images are a good tool to investigate whether the magnetic nanoparticles are completely inside or at least partly outside the nanofibers.

**Figure 5:**
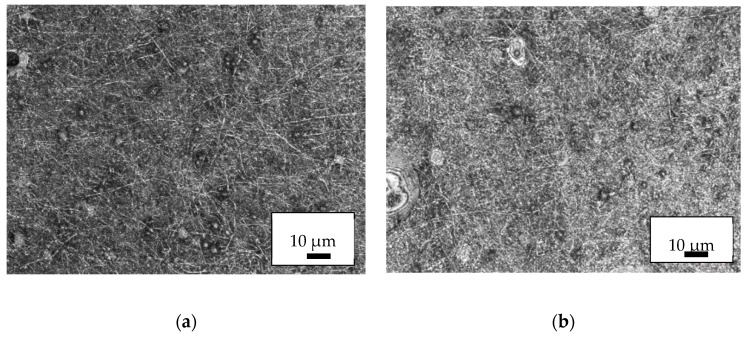

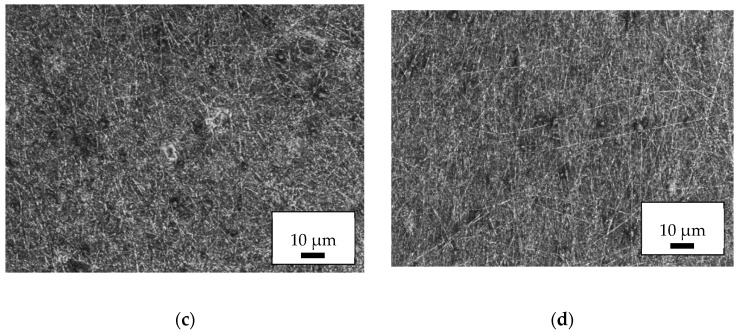
CLSM (confocal laser scanning microscopy) images of the PAN/magnetite nanofiber mats with different polymer:nanoparticle weight ratios: (**a**) 1:0.38; (**b**) 1:0.79; (**c**) 1:1.25; (**d**) 1:1.8.

Next, Figure 5 depicts CLSM images of typical areas of the PAN/magnetite nanofiber mats. All weight ratios of polymer:nanoparticles can be electrospun without problems. No severe variations of the nanofiber mat morphology are visible. The beads, however, are less evenly distributed than in previous experiments with pure PAN and occur less often [31]. This suggests reducing the PAN solid content to 12% in further tests to create a larger amount of well-distributed beads, or to combine the nanofibers with nanomembrane areas instead.

**Figure 6:**
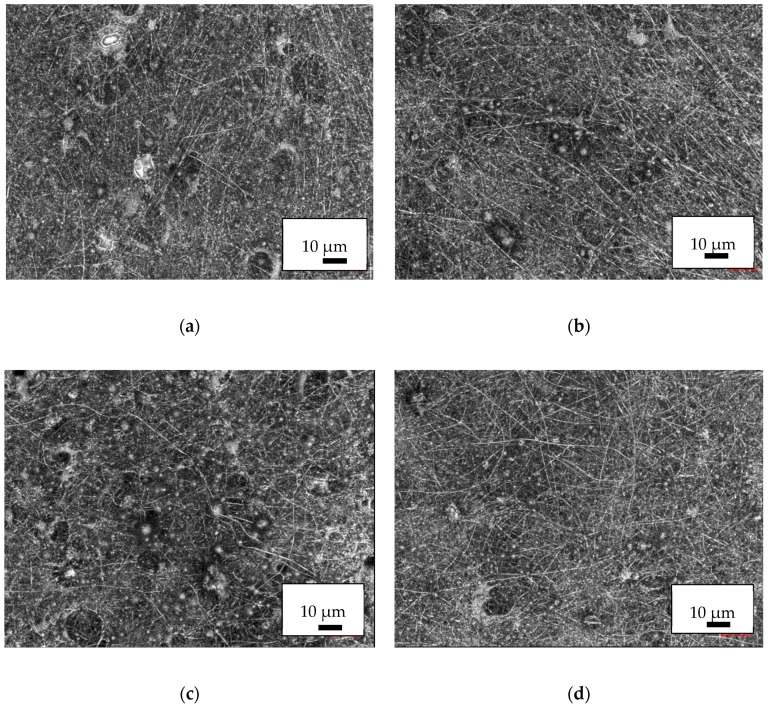
CLSM images of the PAN/diiron nickel tetraoxide nanofiber mats with different polymer:nanoparticle weight ratios: (**a**) 1:0.38; (**b**) 1:0.79; (**c**) 1:1.25; (**d**) 1:1.8.

CLSM images of PAN/diiron nickel tetraoxide nanofiber mats are depicted in Figure 6. On the one hand, all nanofiber mats show larger variations of the fiber distribution, partly even connected with holes in the mats. On the other hand, more and larger beads are visible.

In comparing the nanofiber mats with these magnetic nanoparticles, it would seem a more detailed study is necessary in the future to create a combination of the positive findings of both materials combinations, i.e., a nanofiber mat with well-distributed beads and at the same time without large variations of the morphology. Possible approaches for this are variations of the solid content, the spinning parameters—especially the high voltage— and the nanoparticles.

However, it should be mentionedthat careful investigation of all nanofiber mats by CLSM did not reveal evidence for agglomerations of the nanoparticles outside the polymer matrix. All darker spots found in the light microscopic part of the CLSM were identical with polymer beads in which apparently the nanoparticles formed agglomerations—which is desired to create different magnetic properties in these relatively large objects, as compared to the nanofibers connecting the beads.

**Figure 7:**
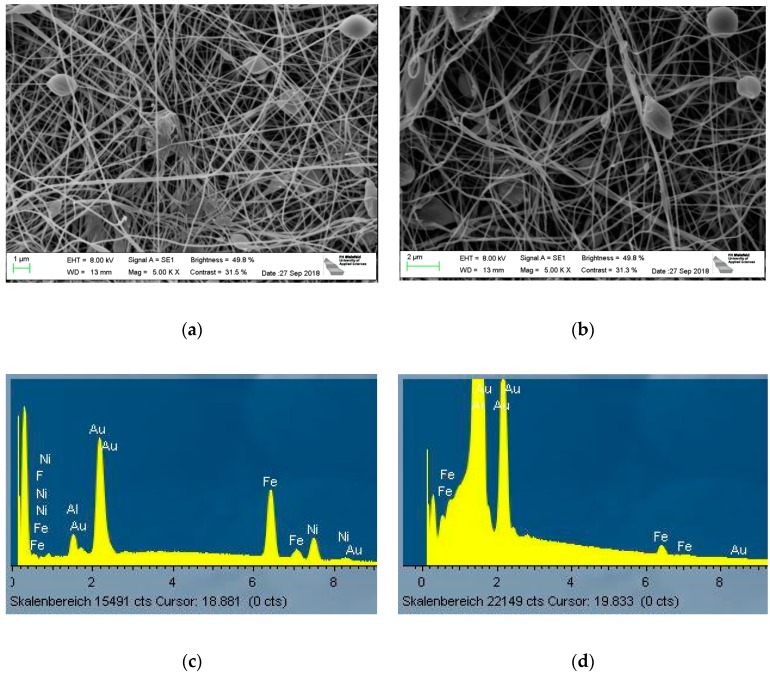
SEM images with a nominal magnification of 5000 x, showing (**a**) a PAN/diiron nickel tetraoxide nanofiber mats with a weight ratio of 1:0.79; (**b**) PAN/magnetite nanofiber mat with a weight ratio of 1:0.79; (**c**) and (**d**) corresponding EDX spectra.

For a closer investigation, Figure 7 shows SEM images of two exemplarily chosen samples. Here the relatively straight, even fibers with some beads are more visible. Their diameters, measured using 50 fibers each, are (133 ± 37) nm for the PAN/diiron nickel tetraoxide nanofiber mat and (121 ± 40) nm for the PAN/magnetite nanofiber mat. Comparable values without significant deviations were found for all nanofiber mats with magnetic nanoparticles examined in this study, while a previous investigation of pure PAN nanofiber mats on nonconductive substrates showed also very similar nanofiber diameters of (144 ± 40) nm [42]. Similarly, the morphologies of the nanofiber mats with and without magnetic nanofibers cannot be distinguished optically [42].

The corresponding EDX spectra are depicted in Figure 7c,d. Besides the gold which was sputtered onto the nanofiber mat to avoid electrostatic charging, the PAN/diiron nickel tetraoxide nanofiber mat clearly shows the elements iron and nickel, while only iron is visible in the PAN/magnetite nanofiber mat.

**Figure 8:**
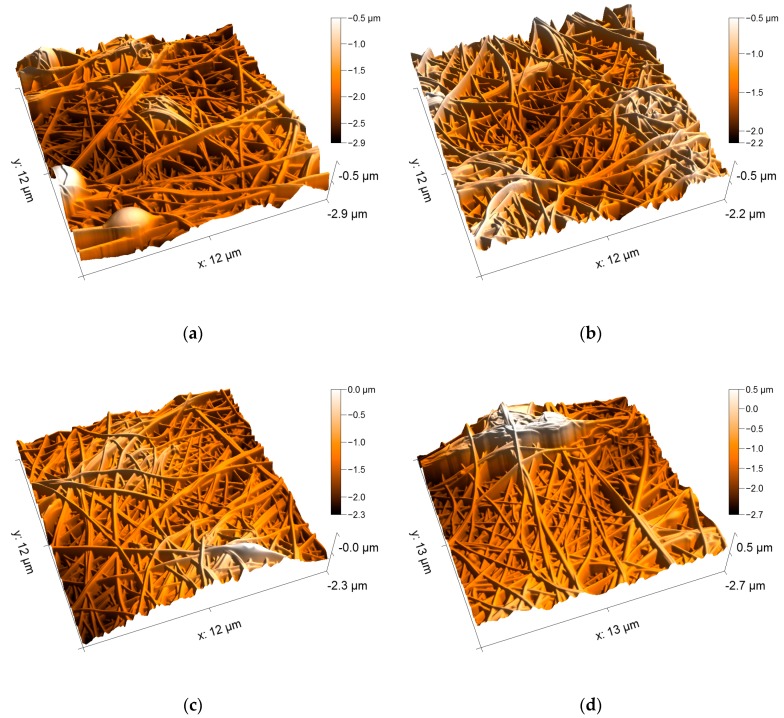
AFM images of the PAN/magnetite nanofiber mats with different polymer:nanoparticle weight ratios: (**a**) 1:0.38; (**b**) 1:0.79; (**c**) 1:1.25; (**d**) 1:1.8.

Figure 8 depicts AFM images of the PAN/magnetite nanofiber mats. Here it becomes clearly visible that on the one hand, all samples consist of even, straight, well-separated fibers. The fiber surfaces are absolutely even, comparable to the fibers visible in Figure 4a, unlike the nanofiber mat with attached nanoparticles, as visible in Figure 4b. Apparently, even with the highest amount of nanoparticles which allowed for electrospinning well-defined nanofiber mats, the nanoparticles are still completely included in the nanofibers.

Comparing the four different nanoparticle contents, no differences in the morphologies are visible. Apparently, even the highest amount of nanoparticles does not disturb fiber formation. This finding can be explained by the significantly different densities of PAN (~1.18 g/cm³) and magnetite (~5.2 g/cm³). The highest weight ratio of 1:1.8 corresponds to a volume ratio of approximately 1:0.4, enabling the nanoparticles to be completely embedded in the polymer matrix. It can be assumed that the weight ratio 1:2.1, which was found to be not spinnable, is the lowest ratio where the polymer could no longer form a matrix around the nanoparticles and thus not form fibers.

**Figure 9:**
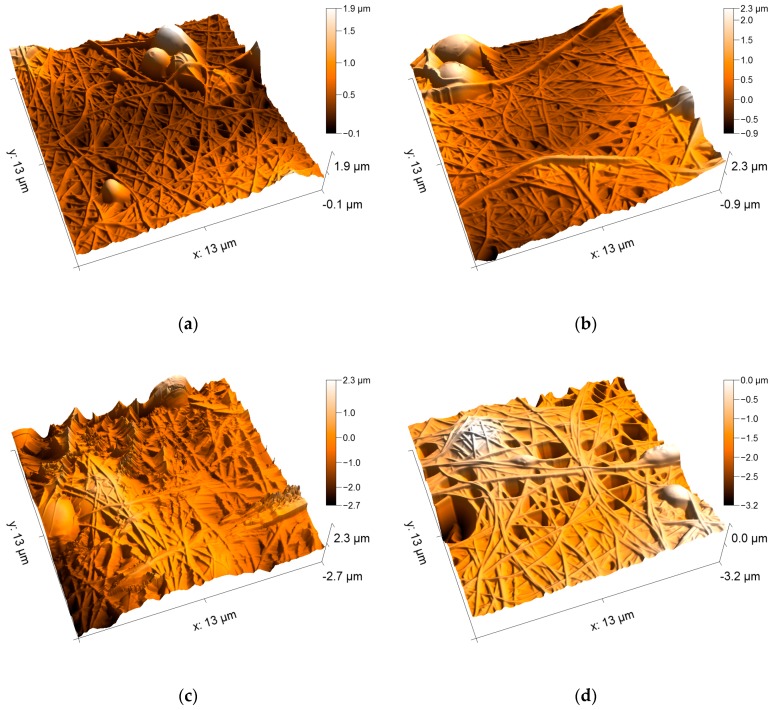
AFM images of the PAN/diiron nickel tetraoxide nanofiber mats with different polymer:nanoparticle weight ratios: (**a**) 1:0.38; (**b**) 1:0.79; (**c**) 1:1.25; (**d**) 1:1.8.

The AFM images of the PAN/diiron nickel tetraoxide nanofiber mats depicted in Figure 9 show a very similar fiber structure for all polymer:nanoparticle weight ratios. Similar to Figure 7, higher nanoparticle contents do not seem to modify the fiber or mat morphology. Here, however, more often beads are visible, as it could be expected from the CLSM images in Figure 6. The bead surfaces are also smooth and do not show any sign of protruding nanoparticles.

The CLSM and AFM images clearly show that now only agglomerations of nanoparticles outside the polymer matrix could be avoided, which could not be expected due to several reports in the literature about chemical pretreatments of such magnetic nanoparticles (without an additional coating) being necessary to avoid agglomerations. In our material system, this purpose could be reached by mechanical separation using ultrasonic treatment. In addition, we have tried embedding magnetic nanoparticles for the first time into nanofiber mats including beads. These beads are densely filled with nanoparticles, as visible from the light-microscopic images of the nanofiber mats showing the beads as dark spots, while the nanoparticles are also completely embedded in the beads and do no protrude, as it can be seen in the AFM images.

## 4. Conclusions

Nanofiber mats were electrospun from polyacrylonitrile blended with different magnetic nanoparticles in various weight ratios. Optical and atomic force microscopic investigations reveal that even in the nanofiber mats with the highest amounts of nanoparticles which could be electrospun without problems, the nanoparticles were completely embedded in the nanofibers. While avoiding undesired agglomerations was often reported in the literature as only possible by chemical modifications of the nanoparticles, here a simple mechanical dispersion by ultrasonic treatment was shown to be sufficient. The beads, which are avoided in many other studies, but are necessary for the planned application of our magnetic nanofiber mats for data storage and processing, were filled with nanoparticles, as desired, without showing protruding nanoparticles. Our study shows that in this way, magnetic nanofiber mats—including beads with different magnetic properties—can be created unambiguously in a one-step electrospinning process.
